# Classification of Brain Tumor from Magnetic Resonance Imaging Using Vision Transformers Ensembling

**DOI:** 10.3390/curroncol29100590

**Published:** 2022-10-07

**Authors:** Sudhakar Tummala, Seifedine Kadry, Syed Ahmad Chan Bukhari, Hafiz Tayyab Rauf

**Affiliations:** 1 Department of Electronics and Communication Engineering, School of Engineering and Sciences, SRM University—AP, Amaravati 522503, India; 2 Department of Applied Data Science, Noroff University College, 4612 Kristiansand, Norway; 3 Department of Electrical and Computer Engineering, Lebanese American University, Byblos P.O. Box 36, Lebanon; 4 Artificial Intelligence Research Center (AIRC), College of Engineering and Information Technology, Ajman University, Ajman 346, United Arab Emirates; 5 Division of Computer Science, Mathematics and Science, Collins College of Professional Studies, St. John’s University, New York, NY 11439, USA; 6 Centre for Smart Systems, AI and Cybersecurity, Staffordshire University, Stoke-on-Trent ST4 2DE, UK

**Keywords:** brain tumor, MRI, diagnosis, vision transformer

## Abstract

The automated classification of brain tumors plays an important role in supporting radiologists in decision making. Recently, vision transformer (ViT)-based deep neural network architectures have gained attention in the computer vision research domain owing to the tremendous success of transformer models in natural language processing. Hence, in this study, the ability of an ensemble of standard ViT models for the diagnosis of brain tumors from T1-weighted (T1w) magnetic resonance imaging (MRI) is investigated. Pretrained and finetuned ViT models (B/16, B/32, L/16, and L/32) on ImageNet were adopted for the classification task. A brain tumor dataset from figshare, consisting of 3064 T1w contrast-enhanced (CE) MRI slices with meningiomas, gliomas, and pituitary tumors, was used for the cross-validation and testing of the ensemble ViT model’s ability to perform a three-class classification task. The best individual model was L/32, with an overall test accuracy of 98.2% at 384 × 384 resolution. The ensemble of all four ViT models demonstrated an overall testing accuracy of 98.7% at the same resolution, outperforming individual model’s ability at both resolutions and their ensembling at 224 × 224 resolution. In conclusion, an ensemble of ViT models could be deployed for the computer-aided diagnosis of brain tumors based on T1w CE MRI, leading to radiologist relief.

## 1. Introduction

Brain tumors (BTs) are characterized by the abnormal growth of neural and glial cells. BTs cause several medical conditions, including the loss of sensation, hearing and vision problems, headaches, nausea, and seizures [1,2]. There exist several types of brain tumors, and the most prevalent cases include meningiomas (originate from the membrane surrounding the brain), which are non-cancerous; gliomas (start from glial cells and the spinal cord); and glioblastomas (grow from the brain), which are cancerous [3,4]. Sometimes, cancer can spread from other parts of the body, which is called brain metastasis [5]. A pituitary tumor is another type of brain tumor that develops in the pituitary gland in the brain, and this gland primarily regulates other glands in the body [6]. Magnetic resonance imaging (MRI) is a versatile imaging method that enables one to noninvasively visualize inside the body, and it is in extensive use in the field of neuroimaging [7]. There exist several structural MRI protocols to visualize inside the brain, but the prime modalities include T1-weighted (T1w), T2-weighted, and T1w contrast-enhanced (CE) MRI. BTs appear with altered pixel intensity contrasts in structural MRI images compared with neighboring normal tissues, enabling clinical radiologists to diagnose them [8].

Several previous studies have attempted to automatically classify brain tumors using MRI images, starting with traditional machine learning classifiers, such as support vector machines (SVMs), k-nearest-neighbor (kNN), and Random Forest, from hand-crafted features of MRI slices [9,10,11,12]. With the rise of convolutional neural network (CNN) deep learning model architectures since 2012, in addition to emerging advanced computational resources, such as GPUs and TPUs, during the past decade, several methods have been proposed for the classification of brain tumors based on the finetuning of the existing state-of-the-art CNN models, such as AlexNet, VGG16, ResNets, Inception, DenseNets, and Xception, which had already been found to be successful for various computer vision tasks [13,14,15,16,17,18,19,20,21,22]. These aforementioned pretrained CNN models based on localized convolutions demonstrated excellent performance in brain tumor classification across different datasets [23,24,25,26]. In a recent study, variational autoencoders, along with generative adversarial networks, were used for synthetic data generation, and ResNet50 was used for tumor classification [18]. In another recent study, transfer learning from VGG16, VGG19, ResNet50, and DenseNet21 models with four different optimization algorithms was implemented, and the authors concluded that ResNet50 performed the best [19]. Despite the tremendous success of CNNs, they generally have inductive biases, i.e., the translation equivariance of the local receptive field. Due to these inductive biases, CNN models have issues when learning long-range information; moreover, data augmentation is generally required for CNNs to improve their performance due to their dependency on local pixel variations during learning.

Lately, attention-based transformer networks [27] have become the de facto models for natural language processing. An adapted version of the transformer for images, the vision transformer (ViT), was proposed in [28], and it seemingly performed superior to CNN models under a huge data regime, as demonstrated by its improved performance when it was trained on the JFT dataset with 300 M images [28]. The ViT models proposed by [28] have less inductive biases due to global patch-based learning, and they can learn more appropriate inductive biases specific to the requirement. In addition, the multi-head self-attention modules in ViT models may facilitate putting better focus on near-tumor regions in MRI images while feature learning compared to CNN models. The usage of ViT models for medical imaging diagnostics is still sparse because ViTs are new, and they require large amounts of data and higher computational resources for training to exhibit their full potential.

Therefore, to fully exploit the power of ViTs, a large amount of data is required, and it may not be possible in medical imaging domains to collect such an amount of data. To overcome this, in [29], several pretrained and finetuned models on ImageNet21k and ImageNet2012 datasets, with various patch sizes and different numbers of multi-head self-attention layers allowing finetuning to a downstream task, are openly available. These approaches have already been found to be successful in a few existing medical imaging diagnostics [30,31,32,33,34]. In [35], the ability of ViTs to classify breast cancer from ultrasound images is presented, and the authors compared the performance of several pretrained and finetuned models and concluded that ViTs performed better than conventional CNNs; in particular, ViT-B/32 achieved superior performance among all the models. In another recent work [36], a ViT-based explainable COVID-19 and pneumonia classification model was developed from chest X-rays and computed tomography images. Another recent work involving a multi-level attention network with the Xception network as a backbone was developed, and the proposed model performed well in tumor classification [25]. Furthermore, ensemble learning generally achieves a better classification accuracy, which has been proven in previous medical imaging diagnosis tasks [37,38,39]. Therefore, in this work, the ability of pretrained and finetuned ViT models, both individually and in an ensemble manner, is evaluated for the classification of meningiomas, gliomas, and pituitary tumors from T1w CE MRI at both 224 × 224 and 384 × 384 resolutions, which, to the best of our knowledge, has not been implemented to date.

## 2. Experimental Methods

This section describes the dataset, the ViT architecture, the computational infrastructure for model training, hyperparameter tuning using the validation set, and testing. The ViT model ensembling and the performance metrics employed are also discussed.

### 2.1. Dataset

**Figure 1:**
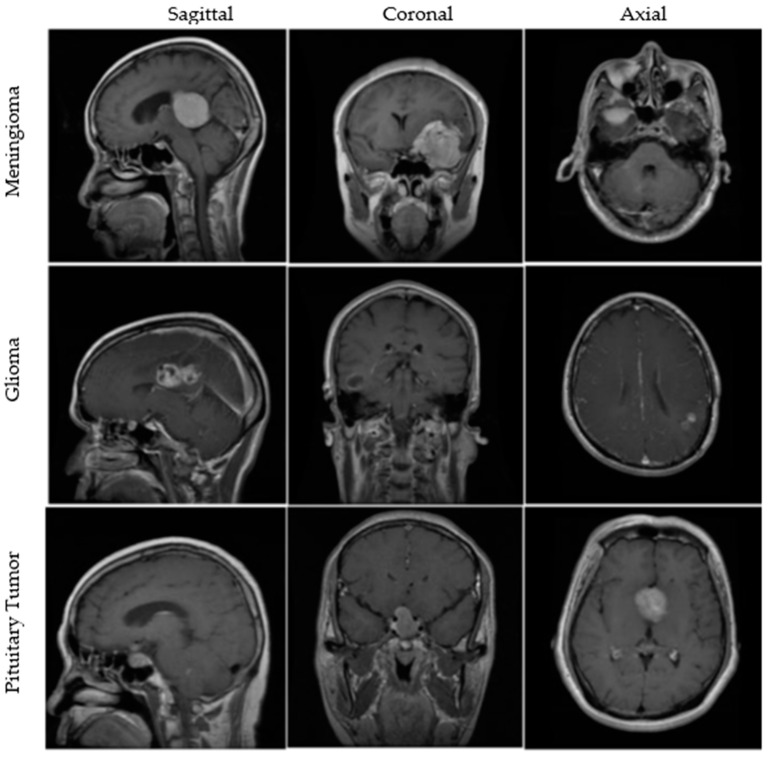
MRI images from the figshare dataset are shown in sagittal, coronal, and axial cut planes for meningiomas, gliomas, and pituitary tumors.

An openly available dataset from figshare consists of 3064 T1w CE MRI slices from 233 patients with meningiomas, gliomas, or pituitary tumors. The images are available in all sagittal, coronal, and axial directions, with spatial resolutions of either 512 × 512 or 256 × 256. More details about the dataset are available in [40,41]. A few MRI images from the dataset are illustrated in Figure 1. Furthermore, brief clinical descriptions about the three types of tumors are given below.

Meningiomas: Meningiomas are mostly benign tumors originating from the arachnoid cap cells and often occur in older-age individuals and females. These tumors account for 13–26% of all intracranial tumors [42].

Gliomas: Gliomas are the most frequent and primary intracranial tumors that are malignant. They represent 81% of all intracranial tumors that can cause significant mortality and morbidity [43].

Pituitary Tumors: Pituitary tumors originate in the pituitary gland and are mostly benign. Since this gland regulates different hormones, tumors present in it may cause severe changes in the body. These tumors contribute to 10–15% of all intracranial tumors [3].

The number of images for each tumor category and the number of images used for training, validation, and testing in a 70:10:20 ratio are described in Table 1.

### 2.2. Vision Transformer

**Figure 2:**
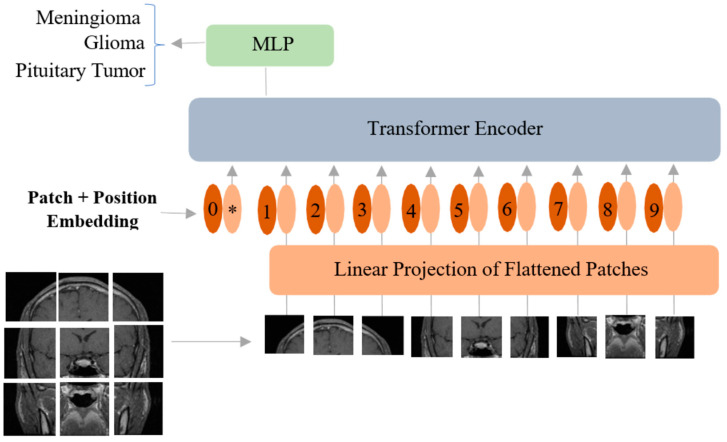
Vision transformer model adopted for classification of brain tumors from MRI. MLP: multilayer perceptron. * is the extra learnable patch embedding to be used by the final classification head.

The ViT proposed by [28] works by treating image patches as words to mimic the original transformer model developed for natural language processing tasks [27]. Although the original transformer model has a combination of both an encoder and a decoder, the ViT model only has an encoder in its architecture. In ViT, the input image I is ${ℛ}^{H\times W\times C}$, and it is divided into N patches of size P×P×C, where $N=\frac{HW}{P^{2}}$ (*H*: height, *W*: width, *C*: number of channels). Afterward, linear embeddings are computed for these flattened image patches, and position embeddings are added to them to keep the patch positional information (Figure 2).

**Figure 3:**
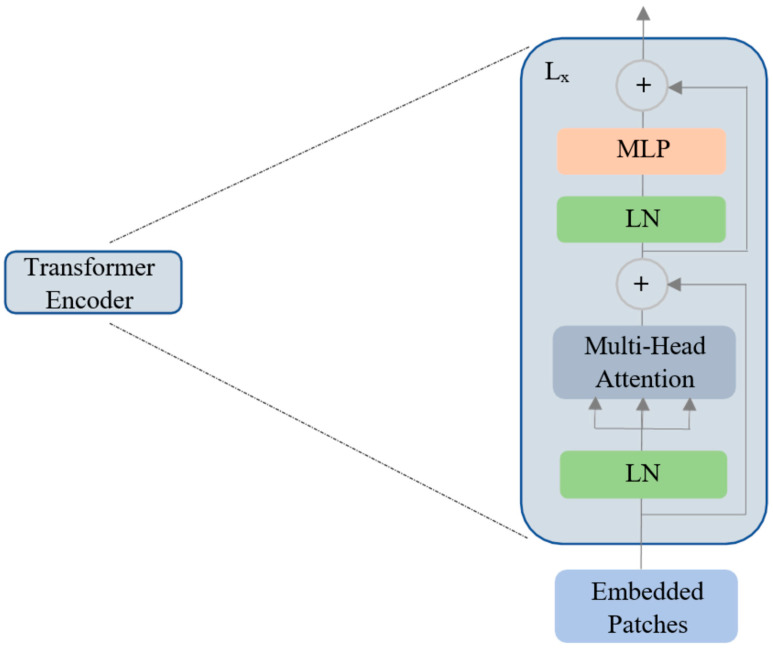
The vision transformer encoder with multi-head self-attention. LN: layer normalization, MLP: multilayer perceptron, L*_x_*: transformer encoder ‘*x*’ at layer L.

An extra learnable patch embedding is added for final classification by a multilayer perceptron (MLP) head. Furthermore, these combined patches and position embeddings are fed to the transformer encoder model, which has alternating layers of multi-headed self-attention and MLP blocks (Figure 3).

In this work, pretrained and finetuned ViT base (B) and large (L) models, B/16, L/16, B/32, and L/32 (16 and 32 indicate square patch size), on ImageNet-21k and ImageNet-1k datasets were used. Hence, the MRI images were resized to the resolutions of 224 × 224 and 384 × 384. Since these pretrained ViT models require three channels in the input and since the MRI slice has a single channel, the same grayscale MRI image is copied into the other two channels.

Similar to [class] in BERT [44], a learnable embedding is concatenated to the sequence of patch embeddings $\left(z_{0}^{0}=I_{class}\right)$. Mathematically, the working principle of ViT is given below in Equations (1)–(4). In Equation (1), $E_{pos}$ is the positional embedding, which is a matrix of learnable parameters; $x_{p}^{N}E$ is the embedding of patch *N,* which is a learnable linear projection; and $z_{0}$ is the output of the linear projection layer. The addition of the position embeddings facilitates the establishment of a certain order in the input image patches. The first block of the transformer encoder layer starts with layer normalization (LN), followed by multi-head self-attention (MSA), and a residual connection follows that produces an output $z_{l}^{\prime }$ at the corresponding layer *l*. The second block also starts with an LN layer, followed by an MLP and a residual connection with output $z_{l},$ as described in Equations (2) and (3). The transformer encoder model is shown in Figure 3. The MLP in the transformer block contains two fully connected layers with Gaussian error linear unit (GELU) nonlinearity. The output of the final transformer encoder layer is $z_{L}^{0},$ which is further layer-normalized as described in Equation (4) to obtain the final latent representation y (with dimension D) of the input image I. The MLP head or the final classification head is attached to this final latent representation (Figure 2) during both pretraining and finetuning.

(1) $$z_{0}=\left[I_{class};x_{p}^{1}E;x_{p}^{2}E;\ldots ;x_{p}^{N}E\right]+E_{pos}\;\;\;\;\;E\in {ℛ}^{(P^{2}.C)\times D},\;\;\;E_{pos}\in {ℛ}^{\left(N+1\right)\times D}$$

(2) $$z_{l}^{\prime }=MSA\left(LN\left(z_{l-1}\right)\right)+z_{l-1}\;\;\;\;\;l=1\ldots L$$

(3) $$z_{l}=MLP\left(LN\left(z_{l}^{\prime }\right)\right)+z_{l}^{\prime }\;\;\;\;\;l=1\ldots L$$

(4) $$y=LN\left(z_{L}^{0}\right)$$

The *MSA* output in the transformer encoder is obtained from the concatenation of several self-attention heads within it. Mathematically, self-attention is described in Equation (5), where *Q*, *K*, and *V* are the query, key, and value matrices obtained after matrix multiplications with $z_{l-1}$, respectively. For example, the *Q* matrix is obtained as $Q=z_{l-1}W_{Q}$, where $Q\in {ℛ}^{\left(N+1\right)\times D}$ and $\;W_{Q}\in {ℛ}^{D\times D}$. Likewise, $K=z_{l-1}W_{K}$ and $V=z_{l-1}W_{V}$. The weights of the matrices $W_{Q}$, $W_{K}$, and $W_{V}$ are learnable. In the self-attention head ($H\in {ℛ}^{\left(N+1\right)\times D}$) given in Equation (5), the product of the query with the key is scaled with the square root of the dimension to avoid the vanishing gradient problem.

(5) $$H=Attention\;\left(Q,K,\;V\right)=softmax\left(\frac{QK^{T}}{\sqrt{D}}\right)V$$

(6) $$MSA\left(Q,\;K,\;V\right)=\left[H_{1},\;H_{2},\ldots ,\;H_{h}\right]\;W_{o}$$

The final output of *MSA* (${ℛ}^{\left(N+1\right)\times D})$ is obtained by passing the concatenation of all self-attention heads through a linear layer as described in Equation (6), where $W_{o}\in {ℛ}^{\left(D\times h\right)\times D}$ is the learnable output transformation matrix, and *h* is the number of self-attention heads. More details about the pretraining and finetuning of the ViT models on larger datasets are described in [28].

### 2.3. Computational Infrastructure

The Google Colab Pro cloud environment, which provides about 25 GB RAM, along with an Nvidia T4 GPU accelerator, was used. The model training, validation, and testing were implemented in TensorFlow 2.8.0, which has *Keras* as a high-level API. The pretrained and finetuned ViT models available in the *vit-keras* module are used by removing the top layer for the downstream task of the three-class classification of brain tumors from the figshare dataset. Custom Python scripts were written where and when necessary.

### 2.4. Model Ensembling

To evaluate the ensemble model for class prediction, the procedures described in Equations (7) and (8) are followed. The softmax outputs of each model (*softmax_i_*) are dot-wise-added and finally divided by the number of individual models (*N*) to obtain the final output (*softmax_e_*) of the ensemble classifier. Two ensembling procedures are evaluated, where the first one is the ensemble of all models at 224 × 224 resolution, and the second ensemble is the combining of all individual models at 384 × 384 resolution.

(7) $$softmax_{e}=\frac{1}{N}\overset{N}{\underset{i=1}{\sum }}softmax_{i}$$

(8) $$final\;class\;prediction=argmax\left(softmax_{e}\right)$$

### 2.5. Performance Metrics

Since a multi-class classification task is carried out, sparse categorical cross-entropy is used as the loss metric, and sparse categorical accuracy is used as the performance metric during training and validation. The confusion matrix and overall sparse categorical accuracy are used as model evaluation metrics during testing. In addition, overall sensitivity and specificity calculated as means of per-class sensitivities and specificities respectively are also used as performance metrics for the ensemble models. The tuned model’s hyperparameters are the optimizer (*RMSprop/Adam/Adadelta*), the learning rate (*lr*), the number of epochs (*ne*), and the mini-batch size (*mbs*). The optimization of the hyperparameters is conducted using the validation set. To calculate the performance metrics on the test set, the hyperparameters that gave the best accuracy values during the 5-fold cross-validation are considered.

## 3. Results

Initially, the image intensities were rescaled to produce values between -1 and 1, which is a requirement for ViT models. During training, all parameters of the ViT models were allowed to be finetuned. For the input image resolution of 224 × 224, the optimized hyperparameters with respect to the validation accuracy were the *Adam* optimizer with *lr* = 0.0001, *ne* = 25, and *mbs* = 16. The B/16 model performed the best at this resolution, with a validation accuracy of 97.83%. Regarding the remainder of the models, their performances at different hyperparameter combinations are given in Table 2, and the best hyperparameters and accuracy values are highlighted.

Similarly, at 384 × 384 resolution, the optimized hyperparameters for the best validation accuracy of 98.64% from the L/16 model were *Adadelta* with *lr* = 0.1, *ne* = 10, and *mbs* = 8. *Adadelta* was solely the best optimizer at this resolution. The optimized hyperparameters and validation accuracies of all other models, B/16, B/32, L/16, and L/32, were 98.10%, 98.04%, and 98.55%. Due to computational constraints, training at 384 resolution was implemented with lower *mbs* values.

The test accuracy values for both the input image resolutions of 224 × 224 and 384 × 384 for all ViT models are given in Table 3. Among all the models, ViT-B/16 performed well, with an overall accuracy of 97.06% at 224 × 224. Similarly, at the resolution of 384 × 384, ViT-L/32 emerged as the single best classifier, with an overall test accuracy of 98.21%. The performance of the average ensembling on the test set is given in Table 4. The ensembling of the models at 224 × 224 resolution resulted in an overall accuracy of 97.71%, and the overall test accuracy of the ensemble model at 384 × 384 resolution was 98.7%. Table 4 also includes overall sensitivity and specificity values for the ensemble model at both resolutions.

**Figure 4:**
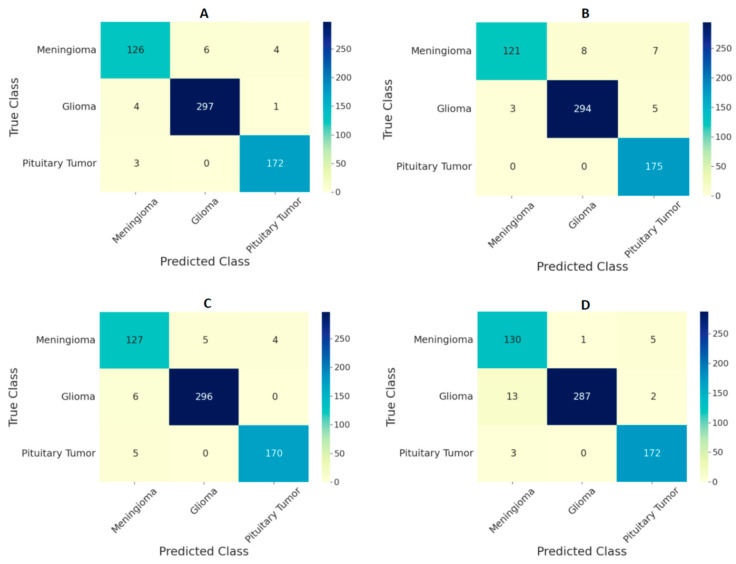
Confusion matrix for classification of three types of tumors on the test set using ViT models (**A**) B/16, (**B**) B/32, (**C**) L/16, and (**D**) L/32 at the image resolution of 224 × 224.

**Figure 5:**
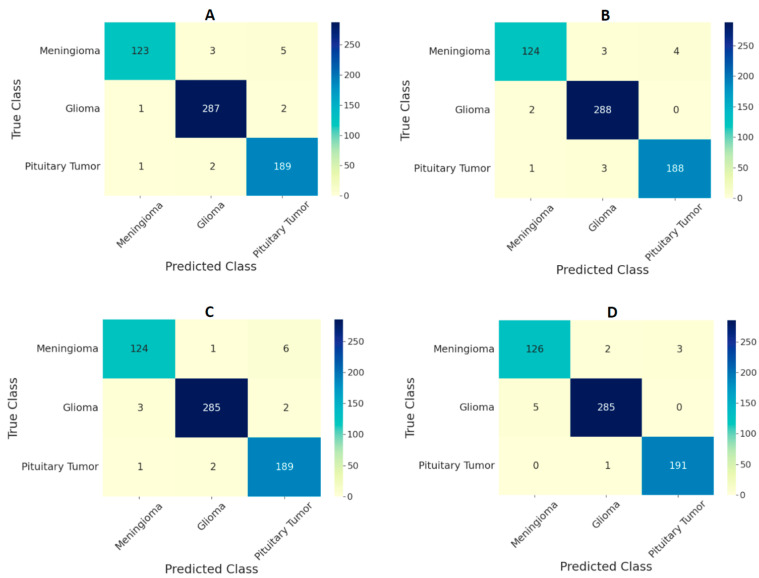
Confusion matrix for classification of three types of tumors on the test set using ViT models (**A**) B/16, (**B**) B/32, (**C**) L/16, and (**D**) L/32 at the image resolution of 384 × 384.

**Figure 6:**
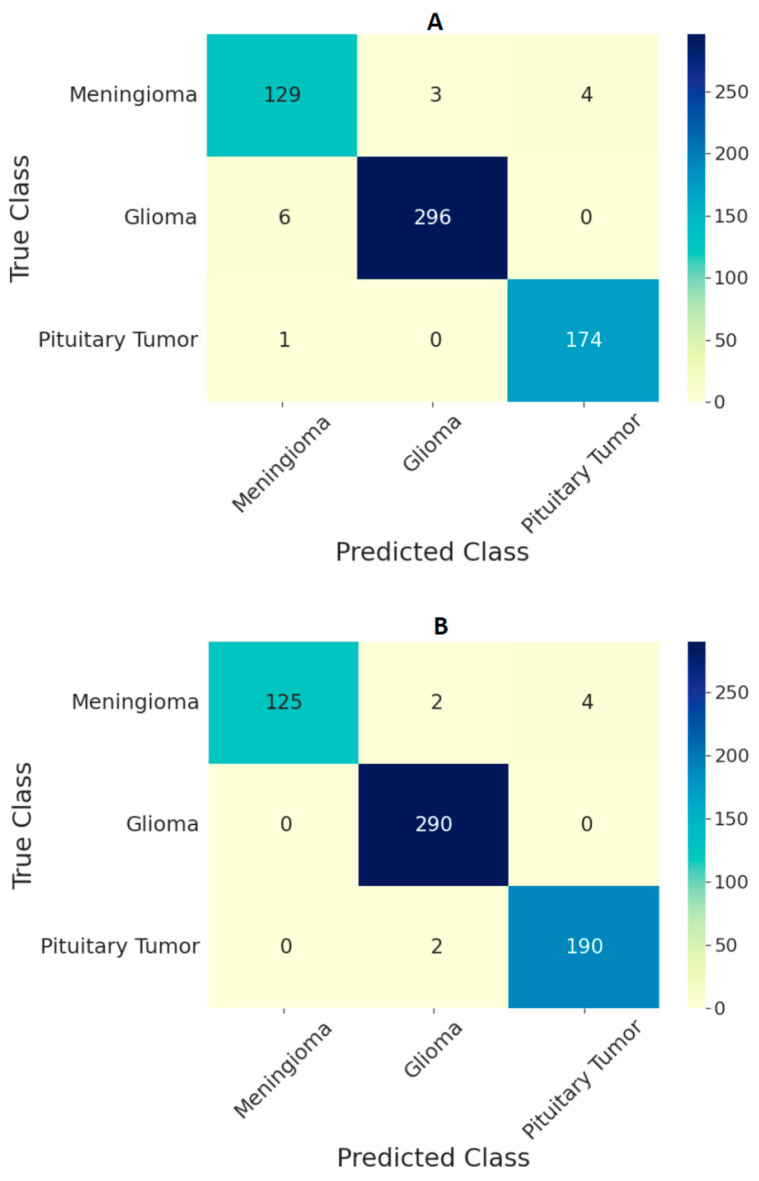
Confusion matrix for classification of three types of tumors on the test set using an ensemble of ViT models B/16, B/32, L/16, and L/32 at (**A**) 224 × 224 resolution and (**B**) 384 × 384 resolution.

The performance of the ViT models on the test set in the form of confusion matrices is given in Figure 4 and Figure 5 for 224 × 224 and 384 × 384 resolutions, respectively. The number of false predictions was higher for meningiomas and gliomas than for pituitary tumors. A similar trend was observed at the two resolutions. However, the number of false predictions was relatively lower at 384 × 384 resolution. Figure 6 shows the confusion matrices for the ensemble model’s performance at both resolutions on the test set. The number of false predictions for the ensemble model at 384 × 384 resolution was eight; moreover, the ensemble model achieved 100% accuracy in the identification of gliomas.

## 4. Discussion

In this study, the ability of pretrained and finetuned ViT models is investigated both individually and in an ensemble manner for a three-class classification of brain tumors, namely, meningiomas, gliomas, and pituitary tumors, from T1w CE MRI. In general, all ViT models demonstrated the ability to classify with validation and test accuracies above 97% during most scenarios (refer to Table 2 and Table 3). Based on the hyperparameter tuning using the validation set, the performance of all the models was good irrespective of the choice of the model hyperparameters, namely, the optimizer, *lr*, *ne*, and *mbs*, which indicates that the ViT models are robust across different hyperparameter settings; however, the *Adadelta* optimizer outperformed all other optimizers at 384 × 384 resolution. Nevertheless, to evaluate the performance of the models on the test set, the models that yielded the highest accuracy values on the validation set were considered, which is the standard procedure. Individual model’s performances on both the validation and test sets were slightly better at the image resolution of 384 × 384 compared to 224 × 224, which could be because the general performance of ViT models is better at higher resolutions, as evaluated by the experiments in [28]. Similarly, the ensemble model’s performance at 384 × 384 was better than that of the ensemble model’s performance at 224 × 224 because average ensembling was used, and the ensemble model’s performance depends on each individual model’s performance in the group.

Comparing the performances of the ViT models in this study with previous studies based on the same dataset given in Table 5, the ensemble of ViTs at 384 × 384 resolution performed better, with an overall test accuracy of 98.7%. Based on the confusion matrices on the test set from all the models at both input image resolutions (Figure 4 and Figure 5), meningiomas had a higher number of misclassifications than gliomas and pituitary tumors, possibly because there could be feature overlapping between the image encodings of meningiomas and gliomas, as well as meningiomas and pituitary tumors. Previous studies have documented a similar trend of misclassification in test set results [19,22]. Our study outperformed all previous studies based on custom CNNs and transfer learning methods, indicating that the pretrained and finetuned ViT models are superior to CNN-based models. The only study that performed marginally better was the CNN-based study in [19]; however, our study was based on ViTs with a different test set, and the number of false predictions was just eight using the ensemble model at 384 × 384 resolution, as shown in Figure 6B.

During training, all the model parameters starting from the patch embedding layer were allowed to be finetuned because, based on a few experiments conducted by freezing the initial layers, including some transformer encoder block layers of the ViT models, the validation and test accuracies were around a couple of percentage points lower than the accuracy values obtained by unfreezing all model parameters. Even though the model’s performance improved at 384 × 384 resolution, training at this resolution was computationally demanding and, hence, implemented in a TPU environment. Furthermore, the performance of the ViTs at the original input image resolution of 512 × 512 may be better, and this hypothesis could be investigated in a high-level computing environment. Furthermore, the cross-validated models from this study can be finetuned for use with other brain tumor datasets. In addition, in a future study, it could be interesting to investigate the ability of other vision transformer variants, such as swin vision transformers [45], data-efficient vision transformers [46], and transformer in transformer models [47], for the brain tumor classification from MRI. A python notebook with the specific code and the cross-validated ViT models pertaining to this study can be provided upon reasonable request.

**Table 5:** Previous related work using figshare dataset and performance comparison in terms of overall accuracy on the test set. ViT: vision transformer.

Work	Method	Image Resolution	Training Data	Accuracy
J. Cheng [40]	GLCM-BoW	512 × 512	80%	91.28%
M.R. Ismael [48]	DWT-2D Gabor	512 × 512	70%	91.90%
A. Pashaei [49]	CNN-ELM	512 × 512	70%	93.68%
P. Afshar [50]	CapsuleNet	128 × 128	-	90.89%
S. Deepak [22]	CNN-SVM-kNN	224 × 224	80%	97.80%
O. Polat [19]	Transfer Learning	224 × 224	70%	99.02%
B. Ahmad [18]	GAN-VAEs	512 × 512	60%	96.25%
N.S. Shaik [25]	MANet	224 × 224	-	96.51%
Present study	Ensemble of ViTs	224 × 224	70%	97.71%
		384 × 384	70%	98.70%

## 5. Conclusions

The performance of the ensemble model at 384 × 384 resolution is on par or better than that of previous CNN models for the classification of brain tumors from MRI, achieving an overall test accuracy of 98.7% and a specificity of 99.4%. Using the same ensemble model, the test classification accuracy for gliomas is 100%. The developed framework is made available publicly here. Therefore, the computer-aided diagnosis of brain tumors from T1w CE MRI using an ensemble of finetuned ViT models can be an alternative to manual diagnoses, thereby reducing the burden on clinical radiologists.

## Figures and Tables

**Table 1:** Figshare dataset showing the number of MRI slices for each tumor category. MRI: magnetic resonance imaging, BT: brain tumor, N: number of images.

BT Type	Total Images	Training	Validation	Testing
Meningioma	708	502	75	131
Glioma	1426	988	148	290
Pituitary Tumor	930	647	91	192
Total (N)	3064	2137	314	613

**Table 2:** Validation accuracy values for different optimizers and hyperparameters for ViT-B/16, ViT-B/32, ViT-L/16, and ViT-L/32 for both input image resolutions of 224 × 224 and 384 × 384. ViT: vision transformer, *ne* = number of epochs, *mbs* = mini-batch size, *lr* = learning rate. B: base, L: large. The hyperparameters optimized for accuracy each ViT model are highlighted in bold at both resolutions.

Resolution	Optimizers and Hyperparameters	Validation Accuracy in Percentage			
		ViT-B/16	ViT-B/32	ViT-L/16	ViT-L/32
**224 × 224**	RMSprop $\{\begin{array}{c} lr=0.0001,\;ne=25,\;mbs=16\\ lr=0.0001,\;ne=20,\;mbs=32\\ lr=0.00005,\;ne=15,\;mbs=32 \end{array}$	$\{\begin{array}{c} 96.20\\ 96.41\\ 97.06 \end{array}$	$\{\begin{array}{c} 97.28\\ 97.01\\ 96.47 \end{array}$	$\{\begin{array}{c} 96.10\\ 96.47\\ 95.92 \end{array}$	$\{\begin{array}{c} 96.20\\ 95.92\\ 95.65 \end{array}$
	Adam $\{\begin{array}{c} lr=0.0001,\;ne=25,\;mbs=16\\ lr=0.0001,\;ne=20,\;mbs=32\\ lr=0.00005,\;ne=15,\;mbs=32 \end{array}$	$\{\begin{array}{c} 97.83\\ 97.55\\ 96.47 \end{array}$	$\{\begin{array}{c} 95.92\\ 96.74\\ 96.74 \end{array}$	$\{\begin{array}{c} 96.82\\ 96.40\\ 96.50 \end{array}$	$\{\begin{array}{c} 97.25\\ 96.20\\ 97.25 \end{array}$
	Adadelta $\{\begin{array}{c} lr=0.1,\;ne=15,\;mbs=16\\ lr=0.1,\;ne=20,\;mbs=32\\ lr=0.05,\;ne=15,\;mbs=32 \end{array}$	$\{\begin{array}{c} 97.25\\ 97.01\\ 97.55 \end{array}$	$\{\begin{array}{c} 96.01\\ 96.01\\ 96.20 \end{array}$	$\{\begin{array}{c} 97.28\\ 97.25\\ 97.55 \end{array}$	$\{\begin{array}{c} 97.28\\ 97.25\\ 96.20 \end{array}$
384 × 384	RMSprop $\{\begin{array}{c} lr=0.0001,\;ne=15,\;mbs=8\\ lr=0.0001,\;ne=10,\;mbs=16\\ lr=0.00005,\;ne=10,\;mbs=8 \end{array}$	$\{\begin{array}{c} 97.31\\ 96.60\\ 97.63 \end{array}$	$\{\begin{array}{c} 97.55\\ 97.21\\ 96.74 \end{array}$	$\{\begin{array}{c} 97.40\\ 96.95\\ 97.60 \end{array}$	$\{\begin{array}{c} 96.51\\ 96.60\\ 97.60 \end{array}$
	Adam $\{\begin{array}{c} lr=0.0001,\;ne=15,\;mbs=8\\ lr=0.0001,\;ne=10,\;mbs=16\\ lr=0.00005,\;ne=10,\;mbs=8 \end{array}$	$\{\begin{array}{c} 97.30\\ 97.54\\ 96.90 \end{array}$	$\{\begin{array}{c} 97.11\\ 96.65\\ 97.01 \end{array}$	$\{\begin{array}{c} 96.82\\ 97.40\\ 97.70 \end{array}$	$\{\begin{array}{c} 97.01\\ 97.40\\ 96.60 \end{array}$
	Adadelta $\{\begin{array}{c} lr=0.1,\;ne=10,\;mbs=8\\ lr=0.1,\;ne=15,\;mbs=16\\ lr=0.05,\;ne=10,\;mbs=8 \end{array}$	$\{\begin{array}{c} 97.10\\ 97.80\\ 98.10 \end{array}$	$\{\begin{array}{c} 98.04\\ 97.83\\ 96.84 \end{array}$	$\{\begin{array}{c} 97.90\\ 97.50\\ 98.64 \end{array}$	$\{\begin{array}{c} 98.55\\ 97.60\\ 98.01 \end{array}$

**Table 3:** Test accuracy values are given in percentages for ViT-B/16, ViT-B/32, ViT-L/16, and ViT-L/32 for both resolutions of 224 × 224 and 384 × 384. ViT: vision transformer, B: base, L: large.

Resolution	Accuracy			
	ViT-B/16	ViT-B/32	ViT-L/16	ViT-L/32
224 × 224	97.06	96.25	96.74	96.01
384 × 384	97.72	97.87	97.55	98.21

**Table 4:** Test accuracy, sensitivity, and specificity values are given in percentages for ensemble classification at (a) resolution of 224 × 224 and (b) resolution of 384 × 384. ViT: vision transformer.

Ensemble Model	Accuracy	Sensitivity	Specificity
All ViT models at 224 × 224 resolution	97.71	96.87	99.10
All ViT models at 384 × 384 resolution	98.70	97.78	99.42

## Data Availability

The data used in the study is publicly available from Figshare at brain tumor dataset.
